# Collared Peccary Wallows are Hubs of Animal Activity and Diversity in a Central American Wet Forest

**DOI:** 10.1002/ece3.70713

**Published:** 2025-02-24

**Authors:** Amanda Eckhoff, Alondra Medina‐Charriez, Megan Zerger, Andrea Romero, Destiny Hackney, T. Mitchell Aide, Kelsey Reider

**Affiliations:** ^1^ Department of Biology James Madison University Harrisonburg Virginia USA; ^2^ Department of Biology Murray State University Murray Kentucky USA; ^3^ Department of Biology; Department of Geography, Geology, and Environmental Science University of Wisconsin‐Whitewater Whitewater Wisconsin USA; ^4^ School of Environment and Natural Resources Ohio State University Columbus Ohio USA; ^5^ Smithsonian Center for Conservation and Sustainability National zoo Conservation Biology Institute Washington, DC USA

**Keywords:** aquatic microhabitat, biodiversity, camera traps, Costa Rica, *Dicotyles tajacu*, La Selva Biological Station, passive acoustic monitoring, *Pecari tajacu*, saíno, vertebrate

## Abstract

Despite research linking peccary wallows to increased amphibian biodiversity in wet tropical forests in Amazonia, wallow use by the broader vertebrate community has been overlooked. We investigated collared peccary (*Pecari* (*Dicotyles*) *tajacu*) activity patterns at wallows and used multiple detection methods to assess wallow use by the vertebrate community in a Central American lowland wet forest in northeastern Costa Rica. We found significantly higher vertebrate activity and diversity at wallows compared to the nearby surrounding understory forest. We documented 13 amphibian, 2 reptile, 11 bird, and 16 nonvolant mammal species, and behaviors including reproduction, drinking, and bathing at wallows. Our observations suggest that wallows can sometimes persist for at least 6 years and are consistently used over that time period by peccaries and breeding amphibians. Our study improves understanding of the ecological importance of collared peccary wallows in the region amid recent changes to Central American peccary populations and ongoing land use and climate shifts.

## Graphical abstract

Collared peccary wallows are used by a diverse assemblage of vertebrates in a Central American tropical wet forest. Wallows had significantly higher vertebrate activity and diversity compared to surrounding forest. Wallow visitors included 13 amphibian, 2 reptile, 11 bird, and 16 mammal species, engaged in behaviors including reproduction, drinking, bathing, and predation.

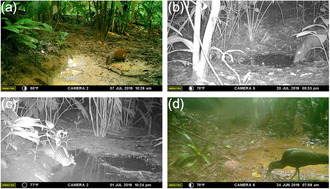

## RESUMEN

A pesar de los estudios que indiacn que los bañaderos de saínos aumentan la diversidad de anfibios en bosques húmedos tropicales en Amazonía, aún no se ha estudiado el uso de bañaderos por la comunidad extensa de vertebrados. Aquí, investigamos los patrones de actividad del saíno acollarado (*Pecari* (*Dicotyles*) *tajacu*) en bañaderos y utilizamos diferentes métodos de detección para asesorar el uso de los bañaderos por la comunidad de vertebrados en un bosque húmedo de baja elevación en el noreste de Costa Rica. Encontramos significativamente mayor actividad y diversidad de vertebrados en los bañaderos en comparación al sotobosque circundante. Documentamos 13 especies de anfibios, 2 reptiles, 11 aves y 16 mamíferos no voladores, así como comportamientos que incluyen reproducción, bebida y baños en los bañaderos. Nuestras observaciones sugieren que los bañaderos pueden persistir a veces hasta 6 años y que son utilizados consistentemente por los saínos y anfibios en reproducción durante ese tiempo. Nuestro estudio aumenta el conocimiento de la importancia ecológica de los bañaderos de saínos acollarados en la región, en medio de los cambios recientes en la población centroamericana de saínos y los cambios en el uso del terreno y el clima.

## 1 Introduction

Many ungulates modify ecosystem‐level processes and habitat availability for other species through behaviors such as trampling, seed dispersal, grazing, nutrient release, rooting, and creating water‐filled depressions such as footprints and wallows (Jones et al. 1994, Hobbs 1996, Baruzzi and Krofel 2017). Wallowing is a widespread behavior among ungulates (Bracke 2011) that results in habitat modification that can affect the occurrence of other organisms. For example, American bison ( *Bison bison* ) wallows create breeding habitat for anurans and other taxa in North American grassland ecosystems (Gerlanc and Kaufman 2003). Ungulate wallows can also be used for bathing and wallowing, and provide drinking water for a wide community of vertebrates. For example, wild pig ( *Sus scrofa* ) wallows were visited by 12 other mammal and bird species in the southeastern United States (Eckert, Keiter, and Beasley 2019).

In the Neotropics two ungulates, collared peccaries (*Pecari* (*Dicotyles*) *tajacu* Linnaeus) and white‐lipped peccaries ( *Tayassu pecari* Link) have a wide geographic distribution and are ecologically important in the tropical lowland forests of Central and South America. They shape understory plant communities and forest dynamics via their effects on seed survival and tree recruitment (Sowls 1997; Beck 2005, 2006), and create and maintain wallows (Sowls 1997).

Peccary wallows are important habitat for many aquatic and semiaquatic amphibian and macroinvertebrate taxa (Zimmerman and Bierregaard 1986; Gascon 1991; Zimmerman and Simberloff 1996; Beck, Thebpanya, and Filiaggi 2010). However, outside of Amazonia, for example, in Central America, there is little information about the amphibian taxa that use peccary wallows. Furthermore, little is known about peccary wallow use by other vertebrate taxa. Observations of nonamphibian vertebrates using peccary wallows have been relegated to personal observations, but many other vertebrate species are assumed to use peccary wallows as habitat (e.g., terrestrial birds, deer, and bats reported to use wallows to forage or drink water in Reyna‐Hurtado et al. 2017). Peccary wallows throughout the Americas may also represent a valuable resource for vertebrate taxa beyond amphibians (e.g., water for drinking and reproduction, foraging, thermoregulation, bathing, and reduction in ectoparasite loads). Thus, the goal of our study was to systematically assess wallow use by the broader vertebrate community in a Central American lowland wet forest.

White‐lipped peccaries have been extirpated from much of their range throughout Central America, and have largely been replaced in terms of their effects on forest and community dynamics by more disturbance‐tolerant collared peccaries (Romero et al. 2013; Reyna‐Hurtado et al. 2017; Thornton et al. 2020). To understand the increasingly important role of collared peccaries in community dynamics, we focused on the role of their wallows in Central America by assessing activity patterns and diversity of the vertebrate community. Our study clarifies the ecological roles and conservation value of these small water bodies for peccaries and other vertebrates that use them. We expected higher activity levels and diversity of vertebrates (amphibians, reptiles, birds, and nonvolant mammals) in the wallows than in the surrounding forest understory.

## 2 Methods

### 2.1 Study Area

The study took place at La Selva Biological Station, a private forest reserve in Heredia Province (10°26′ N, 83°59′ W), northeastern Costa Rica (McDade et al. 1994). La Selva covers 1536 ha of primary, secondary forest, and pastures at 35–137 masl, and averages 4000 mm of rain annually. The climate at La Selva is typical of Atlantic tropical wet forests and is characterized by a bimodal distribution in precipitation, with peaks of > 400 mm/month falling in June–August and October–December (Sanford et al. 1994). Our study spanned June–August 2019 during the wet season. We located small natural water pools throughout La Selva in both old‐growth and secondary forests by attempting to relocate wallows that had been surveyed in 2012 (Reider and Ream 2013), by trail surveys, following peccary tracks, and by asking researchers and technicians to report pools when opportunistically encountered. Pools that had peccary signs including tracks were categorized as peccary wallows, and pools without peccary signs were categorized as “other pools.” Descriptions of peccary wallows from other sites indicate that peccary wallows can occupy portions of broader waterhole habitats (e.g., at Calakmul Biosphere Reserve in Mexico; Delgado‐Martínez, Cudney‐Valenzuela, and Mendoza 2022); however, at La Selva, peccary wallows are frequently isolated, water‐filled depressions. Because our study intended to isolate the effect of peccary‐wallow‐sized pools, we did not include any pools that were connected to flowing water, phytotelmata, or large swamps. Historically, white‐lipped and collared peccaries were present at La Selva, but white‐lipped peccaries were locally extirpated in the 1960s while collared peccaries remain common (Romero et al. 2013).

### 2.2 Bird and Mammal Surveys

Motion‐activated game cameras (Moultrie A‐25) were used to observe bird and nonvolant mammal diversity and behaviors at wallows. Cameras were placed at nine wallows for 5–40 deployment days between June 20, and August 5, 2019. Cameras were installed on natural supports 40–60 cm from the ground and at a distance that provided a clear view of each wallow and its edges.

Three control cameras were paired with three wallow cameras for 26 trap days, the sum of deployment days for the camera pair at a site (Tanwar, Sadhu, and Jhala 2021). The paired control cameras were located facing away from the wallow at a distance of 10–15 m. Analyses of the three paired wallow and control cameras were completed only using data from periods when both cameras were deployed simultaneously (11 days, 11 days, and 4 days, respectively).

Cameras took three photos per trigger event, with no delay between detections. Images taken within 120 s of each other were grouped into sequences. Consecutive sequences of the same species were considered “independent events” if more than 30 min had passed between sequences (Kinnaird et al. 2003). All photos were processed using the online platform Agouti (https://agouti.eu) to identify species to the lowest possible taxonomic group and behaviors. Behaviors included “passing by” (indicating no direct interaction with the wallow), “bathing” (indicating that the individuals entered water or mud in the wallow), “predation,” or a combined category “eating, drinking, or sniffing” defined by seeing the animal's mouth at ground or water level. Predation was defined by seeing an animal with prey in its mouth. We also created a post hoc combined category because some sequences included multiple behaviors: “bathing|eating, drinking or sniffing.”

Weather data were obtained from La Selva's weather station and grouped into half‐hour intervals (https://bixa.tropicalstudies.org/meteoro/default.php). Peccary sequences were separated into day and night to quantify diurnal and nocturnal activity patterns. Night was defined using local astronomical twilight on each camera deployment day as a cut off.

### 2.3 Amphibian Surveys

Passive acoustic monitoring (PAM) and visual encounter surveys (VES) were used to quantify amphibian and reptile diversity at wallows and compare wallows to surrounding understory habitat. We used AudioMoth (LabMaker, Berlin, Germany) acoustic recorders at 13 wallows and 13 paired understory controls which were at random directions and distances from the wallows. The AudioMoths were programmed to record 1 min of audio every 10 min each day simultaneously at each paired wallow and control. Audio files were visualized using the Arbimon Rainforest Connection cloud platform (https://arbimon.rfcx.org/). We identified calls using databases (e.g., Smithsonian Tropical Research Institute, FonoZoo, Macaulay Library), descriptions in Savage (2002), and by consulting experts.

We determined species presence using Arbimon's Pattern Matching function with a low detection threshold (0.15) to decrease the chance of false negatives. All detections were manually validated to eliminate false positives. The resulting occupancy matrix gave the presence and absence of each species for each site per day, and the number of detections per day gave us a metric of relative abundance or relative activity for each species at both wallows and controls (LeBien et al. 2020).

Because not all species or life stages call, we also performed time‐constrained diurnal VES to quantify species richness of amphibians and reptiles at 13 wallows and 6 other pools. VES were done by two independent observers between 10:00 a.m. and 05:00 p.m. Diurnal surveys complement species detected using 24‐h PAM surveys. Diurnal surveys allowed us to include day‐active amphibians and lizard species that do not call, in addition to gathering data on nocturnal amphibian wallow visitors indirectly: Our diurnal surveys were designed to detect eggs and larvae from nocturnal‐breeding amphibians, and adult nocturnal amphibians that remain near the wallow were detected in a water‐conserving posture on leaves in our understory searches. We visited each pool between 1 and 4 times and documented adults, tadpoles, and eggs found within 5 min while searching the pools, up to 50 cm from the wallow edge, and understory up to 2 m above the wallow surface. We identified each individual using descriptions in Savage (2002).

### 2.4 Analysis

R version 4.2.2 (R Core Team 2023) was used for analyses. We compared Shannon diversity calculated from camera trap data for mammals, birds, and reptiles, and separately from PAM data for amphibians, between paired cameras at wallows and controls with a paired *t* test after checking for normality. Shannon diversity from PAM data was calculated as one value for each site using the total number of species occurrences from the entire sampling period at each site. The VES results are reported as a species list.

Activity patterns were characterized from camera trap data for peccaries and for all animals combined using trap rate, a metric that describes the frequency of detections which was calculated for wallow and paired control cameras by dividing the number of independent events by camera deployment days for each location (Bowkett, Rovero, and Marshall 2008). Deployment days are the total number of days a camera was placed out at a location. After checking for normality, a paired *t* test was performed to compare camera trap rates for wallows and control cameras at each location.

## 3 Results

We located 17 collared peccary wallows with surface areas ranging from 0.47 m^2^ to 6.59 m^2^ and average depths (± SD) from 25.2 ± 19 mm to 77.1 ± 8.5 mm (Table 1). One wallow that contained water at the beginning of the study period was dry when we returned after 2 weeks and all others held water throughout the study. Nine wallows were located with prior knowledge of site users, and eight wallows were located during trail surveys. During the same trail surveys, we also located six nonpeccary “other pools” (Table 1), which held water only after two large rainfall events and dried up within 3 days. We focused our vertebrate community surveys on 13 wallows including 11 which had clear signs of recent visitation by collared peccaries. Across all methods, we documented 13 amphibian, 2 reptile, 11 bird, and 16 nonvolant mammal species interacting with or passing along the borders of wallows, and behaviors including reproduction, drinking/eating/sniffing, and bathing at wallows.

**TABLE 1:** Physical measurements of collared peccary wallows and other pools (natural pools without evidence of peccary activity) studied at La Selva Biological Station, Costa Rica.

Location	Type	Length (cm)	Width (cm)	Mean surface area (m^2^)	Mean depth (mm)
CCL350^1,2^	Wallow	297.5 ± 17.7	131 ± 5.7	3.06	37 ± 24
CCL300^1,2^	Wallow	191 ± 12.7	156.5 ± 60	2.35	44.7 ± 20.8
CM1	Wallow	81 ± 11.3	73.5 ± 4.9	0.47	25.2 ± 19
CM2^1,2,3^	Wallow	311 ± 29.7	270 ± 84.9	6.59	51 ± 41.4
SJ700^1,2,3^	Wallow	166.5 ± 62.9	147 ± 58	1.92	68.2 ± 22.2
SSA950^1^	Wallow	223 ± 38.2	116 ± 8.5	2.03	32.9 ± 20
STR5350‐1	Wallow	92	105	0.76	58.3 ± 17.8
STR5350‐2	Wallow	98	87	0.67	35.4 ± 22
STR5350‐3	Wallow	210	97	1.60	53.9 ± 16.7
CC1200^1,2^	Wallow	188 ± 11.3	81 ± 1.4	1.20	55.8 ± 20.9
SCH200‐1^1^	Wallow	173.6 ± 13.6	170.6 ± 12.2	2.33	75.4 ± 24.8
SCH200‐2^1,2^	Wallow	199.2	187	2.93	17.9 ± 17.4
SCH200‐3^1^	Wallow	83.5 ± 56.5	76.5 ± 55.9	0.50	4.3 ± 0.4
SSO1380‐1^1,2^	Wallow	268 ± 17	66.5 ± 12	1.40	38.8 ± 17.6
SSO1380‐2^1,2^	Wallow	119.5	83	0.78	77.1 ± 8.5
LOC1775^1^	Wallow	190	178	2.66	67.2 ± 25.7
LEP450^1,2,3^	Wallow	271.5 ± 21.9	203.5 ± 19	4.34	41.3 ± 16.2
SJ_LOC_1	Other pool	55	20	0.09	4.6 ± 0.8
SJ_LOC_2	Other pool	35	25	0.07	3.4 ± 1.8
SJ_LOC_3	Other pool	20	15	0.02	4.5 ± 1.1
SSA450	Other pool	50	35	0.14	34.3 ± 14.1
SJ1225	Other pool	93	70	0.51	32.9 ± 17.7
SJ1196	Other pool	202	79	1.25	47.9 ± 28.7

*Note:* Length and width measurements are given as mean ± SD when wallows were repeatedly measured, typically once before and after a rainfall event. Measurements without SD are from a single visit to that location. Other pools were only found after large rainfall events and thus do not have repeat measurements for size and length, width, or area. Mean surface area was estimated using the mean length and width to calculate the area of an ellipse. All depth measurements are mean ± SD. All locations were surveyed for amphibians and reptiles using diurnal VES. Wallow locations with 1: (*N* = 13) were also included in the PAM surveys; locations with 2: (*N* = 9) had camera traps at the wallow to survey nonamphibian vertebrate diversity; and locations with 3: (*N* = 3) had paired wallow and control camera traps to compare nonamphibian diversity and activity patterns in the two habitat types.

Using PAM, we detected 12 anuran species in total, and 11 of those were detected on wallows (Table 2). Amphibian Shannon diversity was significantly higher at wallows than understory reference plots using PAM (for diversity calculated for each wallow and control: *t* = 3.625, *p* = 0.005). Using VES, we detected six anurans at wallows including three aquatic‐breeding treefrogs ( *Agalychnis callidryas* , adults, eggs, and tadpoles; *Smilisca baudinii* adults, eggs, and tadpoles; and *Cruziohyla sylviae* adult) and two reptiles (stream anole *Anolis oxylophus* and white‐lipped mud turtle *Kinosternon leucostomum* ) in wallows (Table 3). *Agalychis callidryas* (red‐eyed treefrog) was the most common aquatic‐breeding species detected and was found in nine different wallows along with evidence of successful breeding (presence of egg masses and/or tadpoles) in seven wallows (Table 4). Three aquatic‐breeding amphibian species ( *Cruziohyla sylviae* , *Smilisca baudinii*, and *Smilisca phaeota* ) were detected only at wallows. No amphibians were detected during VES at other pools.

**TABLE 2:** Presence and absence of amphibian species at wallows and controls using passive acoustic monitoring methods.

Species	CCL300		CCL350		CM		SJ700		SSA		STR		SCH1		SCH2		SCH3		SSO1		SSO2		CC1200		LOC	
	W	C	W	C	W	C	W	C	W	C	W	C	W	C	W	C	W	C	W	C	W	C	W	C	W	C
*Rhinella marina*	—	—	X	X	—	—	—	—	X	X	X	X	—	—	—	—	—	—	X	NA	X	NA	—	—	X	X
*Hyalinobatrachium valerioi*	X	X	X	X	X	X	X	X	X	X	X	X	X	X	X	X	X	X	X	NA	X	NA	X	X	X	X
*Craugastor fitzingeri*	X	X	X	X	X	X	X	—	X	X	—	X	—	—	—	—	—	—	X	NA	X	NA	X	X	—	X
*Oophaga pumilio*	X	X	X	X	X	X			X	X	X	X	X		X	X	X	X	X	NA	X	NA	X	X	X	—
*Diasporus diastema*	X	X	X	X	X	X	X	X	X	X	X	X	X	X	X	X	X	X	X	NA	X	NA	X	X	X	X
*Agalychnis callidryas*	X	—	X	—	X	—	—	—	X	X	X	X	X	X	X	—	—	—	X	NA	X	NA	—	—	X	X
*Hypsiboas rufitelus*	X	—	—	—	—	—	—	—	X	—	—	—	X	—	—	—	X	X	X	NA	X	NA		—	X	—
*Smilisca baudinii*	—	—	—	—	X	—	—	—	—		—	—	X	—	—	—		—	—	NA	—	NA	—	—	—	—
*Smilisca phaeota*	—	—	—	—	X	—	—	—	—	—	—	—	—	—	—	—	—	—	—	NA	—	NA	—	—	—	—
*Leptodactylus melanonotus*	—	—	—	—	—	X	—	—	—	—	—	—	—	—	—	X	—	—		NA	—	NA	—		—	—
*Leptodatylus poecilochilus*	X	—	—	—	—	—	—	—			—	—	X	—	X	—			—	NA	—	NA		—		—
*Leptodactylus savagei*	—	—	—	—	—	—	—	—	X	X	—	—	—	—	X	—	X	X	X	NA	—	NA	—	—	—	—

*Note:* X = Species detected on at least 1 day; — = not detected on any days; and NA indicates that the location did not have a paired AudioMoth. Occurrence records from wallows include species likely calling from nearby leaf litter and stream habitats such as the poison frog *Oophaga pumilio* , direct‐developing leaf litter frog *Craugastor fitzingeri* , and glassfrog *Hyalinobatachium valerioi*.

**TABLE 3:** Presence and absence of amphibian and reptile species observed at wallows using Visual Encounter Surveys according to their life stage (egg, tadpole, and adult).

Species	Wallows	Eggs	Tadpoles	Adults
*Agalychnis callidryas*	CCL300	—	X	—
	SSO1	X	X	—
	SSO2	X	X	—
	LEP450	—	X	—
	SSA950	—	X	—
	CCL350	—	X	—
	LOC1775	X	X	—
*Smilisca baudinii*	SCH3	X	X	—
*Oophaga pumilio*	CC1200	—	—	X
*Cruziohyla sylviae*	SJ700	—	—	X
*Anolis oxylophus*	CCL350	—	—	X
	LOC1775	—	—	X
*Kinosternon leucostomum*	SSA950	—	—	X

*Note:* X = Present, — = not detected.

**TABLE 4:** Complete species list for amphibians and reptiles found at collared peccary wallows by VES and PAM methods.

Amphibians
*Rhinella marina* *
*Hyalinobatrachium valerioi* *
*Craugastor fitzingeri* *
*Oophaga pumilio* ^*+^
*Diasporus diastema* *
*Agalychnis callidryas* ^*+^
*Cruziohyla sylviae* ^+^
*Hypsiboas rufitelus* *
*Smilisca baudinii* ^*+^
*Smilisca phaeota* *
*Leptodatylus poecilochilus* *
*Leptodactylus savage* *

Reptiles
*Anolis oxylophus* ^+^
*Kinosternon leucostomum* ^+^

*Note:* *Indicates species was present in PAM and + indicates species was present in VES.

**FIGURE 1:**
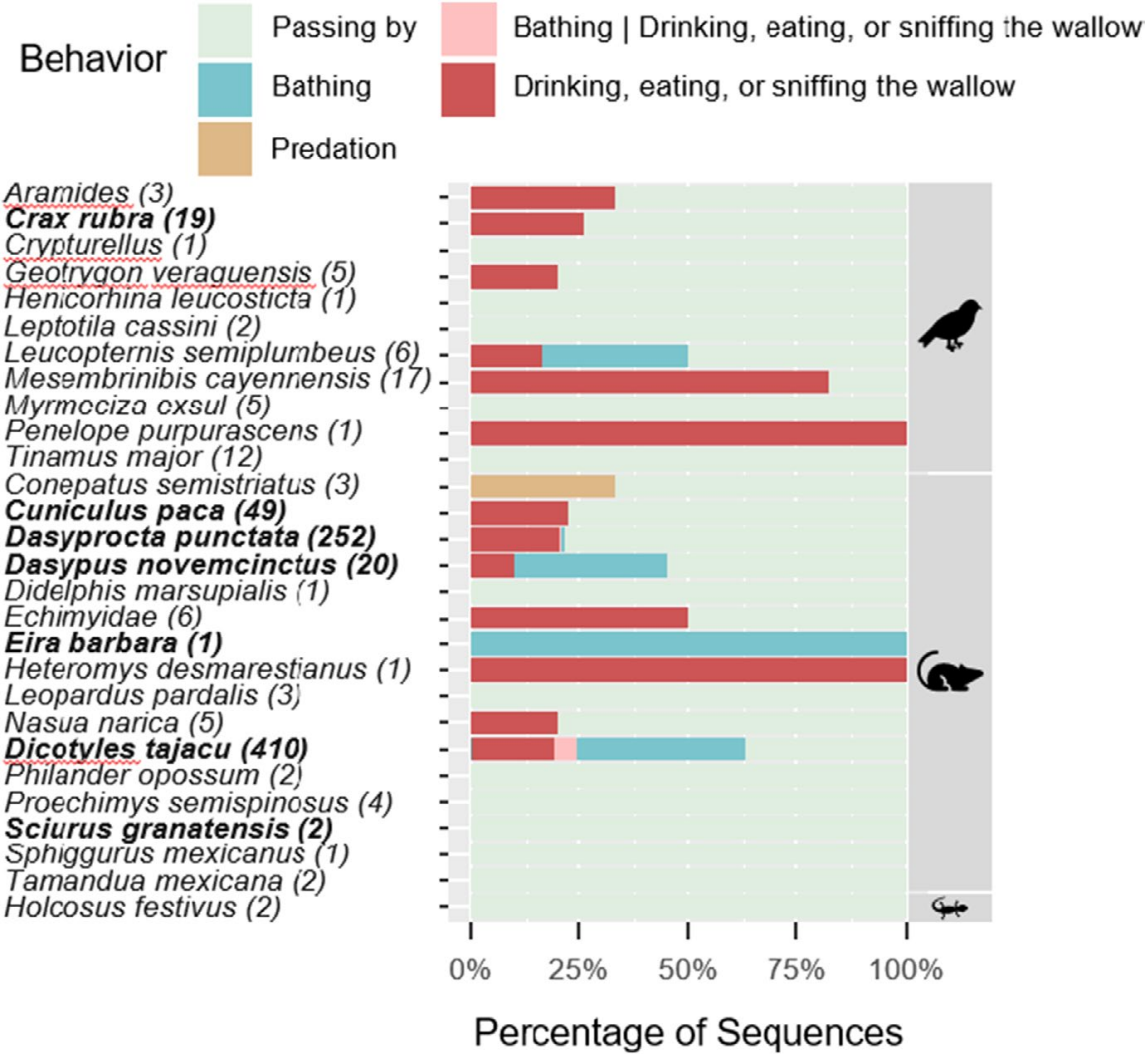
Taxonomic diversity of species and behaviors observed at collared peccary wallows using camera traps. Numbers in parentheses represent the total number of sequences of that species at wallows. Bold text indicates species that were also observed at controls. The | indicates that more than one behavior was observed in a single sequence. The symbols represent the classes Aves, Mammalia, and Reptilia, respectively. Bird families detected at wallows include Accipitridae (2 species), Columbidae (2 species), Cracidae (2 species), Rallidae, Thamnophilidae, Threskiornithidae, Tinimidae (2 species), and Troglodytidae. Mammal families included Cuniculidae, Dasypodidae, Dasyproctidae, Echimyidae, Erethizontidae, Felidae, Heteromyidae, Mephitidae, Mustelidae, Myrmecophagidae, Procyonidae, Sciuridae, and Tayassuidae.

**FIGURE 2:**
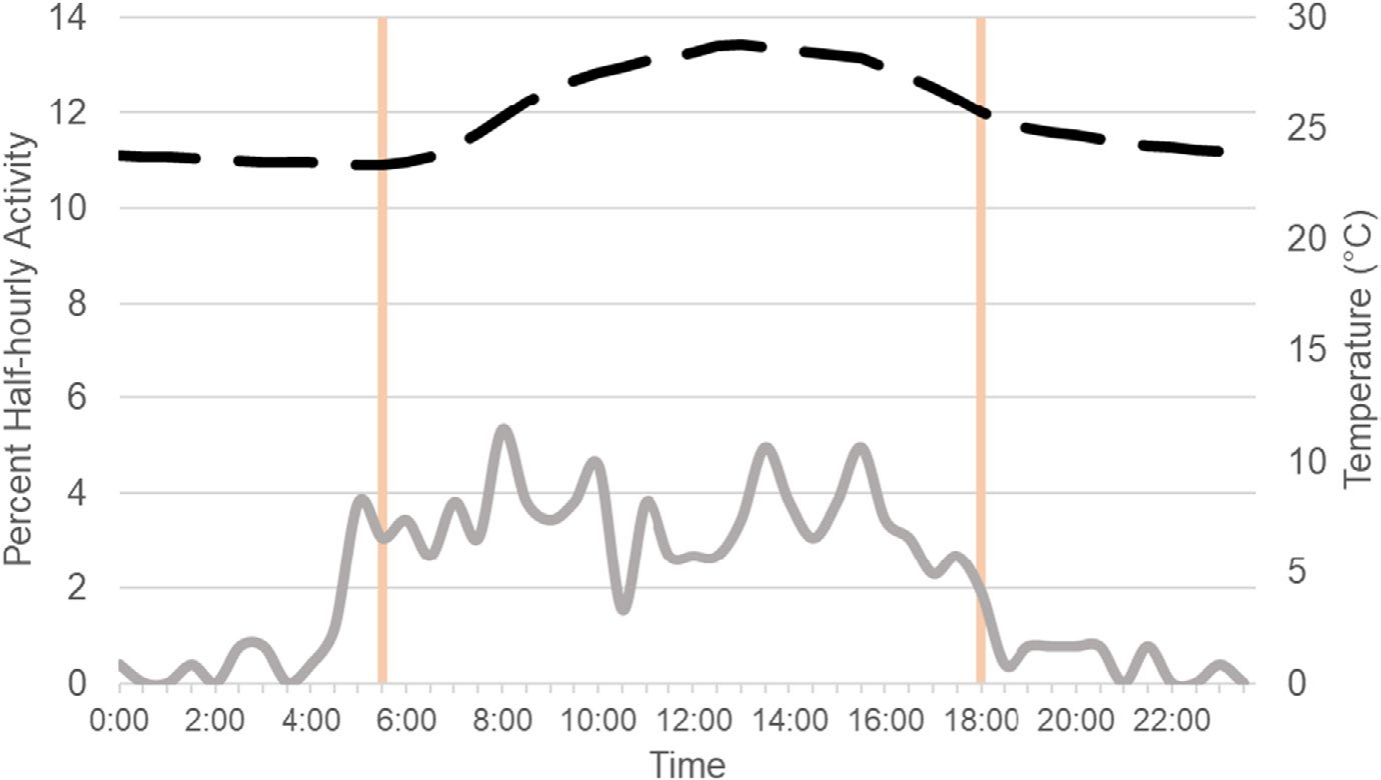
Peccary activity patterns at wallows (percent half hourly activity). The dotted line represents the mean temperature throughout the day, and the vertical lines are median sunrise and sunset for the study period. Air temperature was obtained from La Selva's weather station for the appropriate date range and grouped into half‐hour intervals (https://bixa.tropicalstudies.org/meteoro/default.php).

Camera traps recorded 624 independent events. Eleven bird species and 16 nonvolant mammal species were observed by camera traps at wallows (Figure 1). One lizard (*Holcosus festivus*, Central American whiptail, family Teiidae) was also registered by camera near wallows. Locations with paired cameras had a significantly higher camera trap rate at wallows than paired controls for independent events of all species (*t* = −4.973, *p* = 0.038), but did not differ for peccaries alone (*t* = −0.079, *p* = 0.944). Peccary activity was strongly diurnal (Figure 2) but there were 15 nocturnal independent events of peccaries.

Wallow Shannon's diversity index for nonamphibians was significantly higher on wallows than controls (*t* = −19.829, *p* = 0.003). *Pecari tajacu* (collared peccary) and *Dasyprocta punctata* (Central American agouti) were the most frequently observed species at wallows by number of sequences. All bird (*N* = 1) and mammal species (*N* = 6) detected by control cameras were also detected by wallow cameras.

**FIGURE 3:**
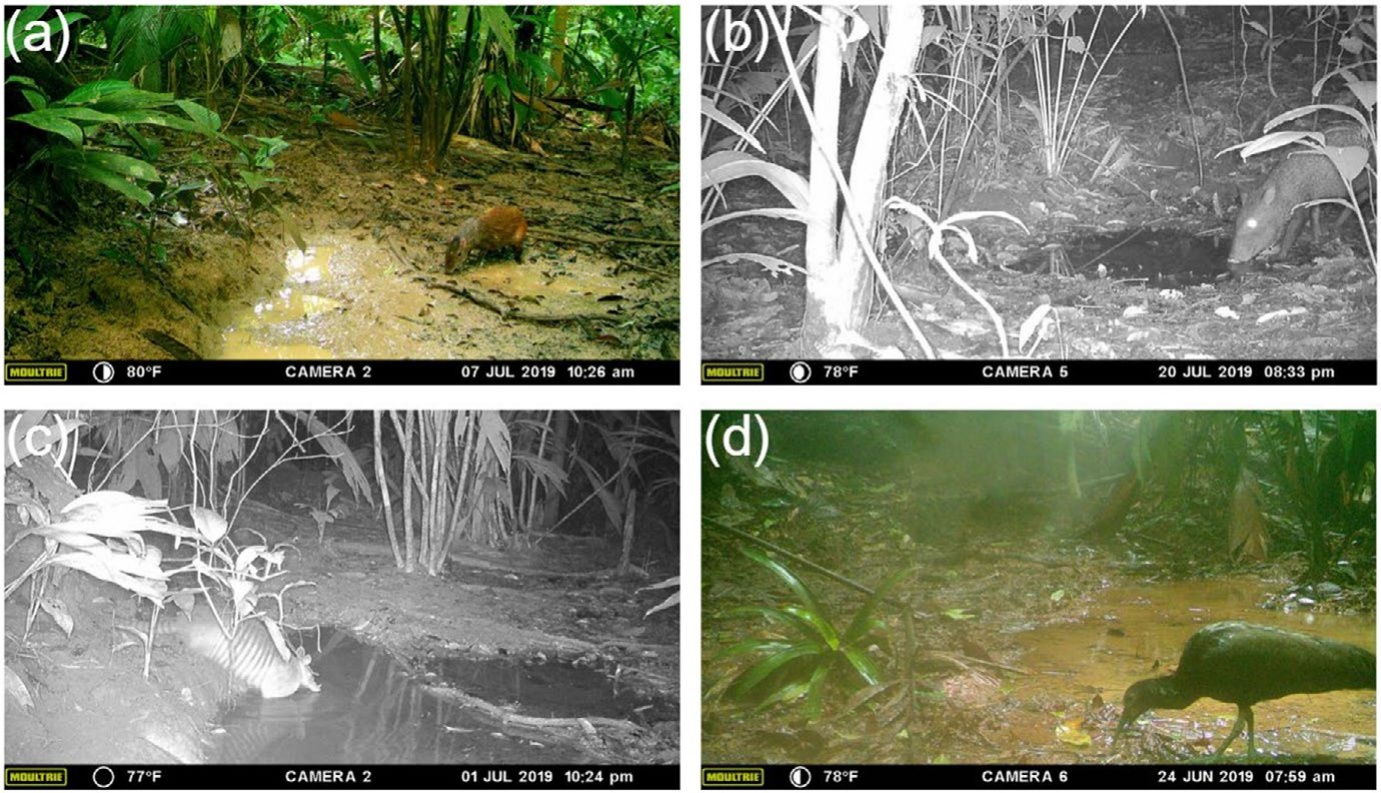
Common species observed at collared peccary wallows. (a) *Dasyprocta punctata* (Central American agouti) eating, drinking, or sniffing; (b) *Pecari* (*Dicotyles*) *tajacu* (collared peccary) at night eating, drinking, or sniffing; (c) *Dasypus novemcinctus* (nine‐banded armadillo) bathing; and (d) *Mesembrinibis cayennensis* (green ibis) eating, drinking, or sniffing.

The most common behaviors for all bird and mammal species were “bathing” and “drinking, eating, sniffing.” Intraspecific interactions such as predation were rarely observed. Some species, such as great tinamou ( *Tinamus major* ) and Tome's spiny rat ( *Proechimys semispinosus* ), only ever passed by the wallow, while some, including the crested guan ( *Penelope purpurascens* ) and green ibis ( *Mesembrinibis cayennensis* ), typically interacted directly with the wallow (Figure 3). The majority of sequences with collared peccaries included a behavioral interaction directly with the wallow, “drinking, eating, sniffing,” “bathing,” or both in the same sequence.

## 4 Discussion

Our study used a combination of camera traps, visual surveys, and acoustic monitoring to assess wallow microhabitat use by a vertebrate community in the Caribbean lowlands of Costa Rica. We documented a diverse assemblage including 42 species of amphibians, reptiles, birds, and nonvolant mammals using wallows for reproduction, bathing, drinking, and eating. Our results extend earlier observations from Amazonia suggesting peccary wallows provide important habitat for amphibians (Zimmerman and Bierregaard 1986; Beck, Thebpanya, and Filiaggi 2010) to Central American lowland forests. We also provide systematic evidence that collared peccary wallows should be viewed not just as important habitat for pond‐breeding amphibians, but for a taxonomically broad vertebrate community. The higher activity and diversity we quantified at wallows compared to the surrounding forest suggests that these small aquatic habitats supply important resources that attract a broad range of vertebrates, even during the wet season in a wet tropical forest.

Beck, Thebpanya, and Filiaggi (2010) found that white‐lipped peccary wallows hold water longer than other natural ponds in the Amazon dry season, making wallows a more predictable water source than other natural pools. Ringler, Hödl, and Ringler (2015) demonstrated that simply supplementing the forest understory with artificial pools similar in size to peccary wallows increased a pool‐breeding amphibian population, suggesting that pool availability may be limiting for some species. Our results provide some support for the hypothesis that peccary wallows supplement pool availability in important ways: We located only a few small other pools without peccary signs, and other pools did not hold water as long as peccary wallows. One of the wallows we studied dried during the study period, whereas all six other pools dried shortly after rainfall. The presence of persistent pools like peccary wallows may become more important if they hold water into drier periods when other pools may become less available. Although our trail searches may have missed additional wallows and other pools away from trails, the presence of peccary wallows should have a stabilizing effect on the availability of small pool habitats throughout the forest understory. Standardized surveys across the forest including during dry periods would allow for a more robust comparison of peccary wallow and other pool density across the forest, and experimental manipulation of pools *sensu* Ringler, Hödl, and Ringler (2015) could reveal if pool availability influences the occurrence or population sizes of organisms.

We also documented another dimension of wallow longevity: Four of the wallows studied were previously documented as in use by peccaries in June–July 2012 (Reider and Ream 2013). Our repeat observations of four of the same wallows 6 years apart indicate collared peccary wallows persist and remain actively used by many species for years, likely due to deepening and soil compaction from repeated peccary wallowing. Wallow longevity may make wallows a reliable resource for the organisms that use them, such as the large‐bodied treefrogs (*Cruziohyla silvae* and *Agalychnis callidryas* ) we observed perched at and breeding in multiple wallows, and Stream Anoles ( *Anolis oxylophus* ), at multiple wallows in both 2012 (Reider and Ream 2013; Ream and Reider 2013) and 2019. Sustained use suggests wallow microhabitats may be part of a natural history strategy of these species rather than chance observations of wayward or outlier individuals. As suggested by Beck, Thebpanya, and Filiaggi (2010), the presence of persistent pools like wallows may enhance connectivity between different habitats for some anurans, and our study suggests that wallows may also serve a similar function for reptile species and other vertebrates. Future studies should use capture–mark–recapture methods to determine if individual animals are wallow residents or are instead using wallows as stopovers during dispersal.

Independent of water body creation by peccaries, specifically, our results suggest that persistent lentic waterbodies on the forest floor serve as point resources that concentrate the activity of vertebrates even in a wet environment during the wet season. Because wallows persist for long periods and are used by many different species, persistent pools likely facilitate interspecific interactions and contribute to overall habitat heterogeneity and diversity in Central American lowland wet forests along with other types of small, persistent pools (e.g., Delgado‐Martínez, Cudney‐Valenzuela, and Mendoza 2022; Perera‐Romero et al. 2021). Individuals may learn the location of persistent pools and visit them repeatedly. Camera traps recorded predators (e.g., ocelot) passing by wallows, and a predation attempt by a skunk ( *Conepatus semistriatus* ) on an unknown species. The cameras recorded many mammal and bird species interacting directly with wallows such as a bathing tayra ( *Eira barbara* ), semiplumbeous hawk ( *Leucopternis semiplumbeus* ), and nine‐banded armadillo ( *Dasypus novemcinctus* ). A lowland paca (*Cuninculus paca*) pair that may have been a mother and offspring repeatedly visited a wallow over the entire camera deployment period and were frequently eating, drinking, or sniffing at the wallow. Longer camera deployments could reveal additional species (e.g., bats, deer, and tapirs) and ecological interactions at collared peccary wallows.

Large‐bodied aquatic‐breeding hylid treefrogs *Cruziohyla sylvae*, S*milisca baudini*, *and S. phaeota* were detected only at wallows by all methods. Similarly, *Hypsiboas rufitelus* and *Agalychnis callidryas* were detected by PAM more often at wallows than in the understory. Detections of two pond‐breeding anurans by PAM at the understory reference plots may be purely methodological: Calls of these species detected in the understory were likely the same individuals detected at a much higher amplitude at wallows. Future PAM studies should separate paired devices by at least 100 m.

The use of small, isolated peccary wallows as habitat by aquatic‐breeding hylid treefrogs is interesting because many of these species are typically associated with open‐water swamps that are several orders of magnitude larger than wallows (Donnelly and Guyer 1994). Notably, absent from peccary wallows were the abundant, smaller‐bodied hylids in the pond‐breeding assemblage in the region, such as *Dendropsophus ebraccatus* and *Scinax elaeochrous* (Donnelly and Guyer 1994). We suspect their absence reflects real species differences such as habitat suitability and dispersal ability rather than seasonal effects because we sampled during the reproductive period of hylid frogs at this site (Donnelly and Guyer 1994). Peccary wallows likely differ substantially from open‐water swamps in terms of environmental characteristics, ecological communities (e.g., algae, emergent plants, macroinvertebrates, predators, etc.), and disturbance regimes likely associated with the frequency of visits by peccaries themselves. Future work could directly compare peccary wallow assemblages to swamps and other habitats such as other small forest floor pools and tree holes.

Peccary activity at wallows was strongly diurnal, but night activity made up 4% of total peccary sequences. Romero et al. (2013) made nocturnal observations of peccaries during walking transects which usually consisted of nonactive sleeping groups. In contrast, our study reveals that peccaries at La Selva occasionally actively bathe in, and eat/drink/sniff at, wallows at night. Carrillo, Saenz, and Fuller (2002), Peterson et al. (2021); Peterson, Jorge, and Keuroghlian (2023), and Hofmann et al. (2016) suggested wallow visitation and nocturnal activity are important for white‐lipped peccary thermoregulation and may help the species cope with climate change, which may also be important for collared peccaries. In addition, the presence of wallows may provide thermoregulatory opportunities for the other species that we observed bathing or encountered in the pools.

The decline and extirpation of white‐lipped peccaries underscore the importance of understanding the ecological roles of peccaries in Central America. Collared peccary wallows are hotspots of diversity and activity for other vertebrates. As land use and climate change alter habitat and community dynamics, collared peccary wallows may represent increasingly valuable microhabitats for a diverse community of vertebrates.

## Author Contributions

**Amanda Eckhoff:** data curation (supporting), formal analysis (lead), investigation (equal), methodology (equal), visualization (lead), writing – original draft (supporting). **Alondra Medina‐Charriez:** formal analysis (equal), investigation (lead), methodology (equal), writing – original draft (supporting). **Megan Zerger:** data curation (equal), investigation (supporting), validation (supporting), writing – review and editing (supporting). **Andrea Romero:** data curation (supporting), investigation (supporting), validation (supporting), writing – review and editing (supporting). **Destiny Hackney:** investigation (equal), methodology (supporting), writing – review and editing (supporting). **T. Mitchell Aide:** investigation (supporting), methodology (equal), writing – review and editing (equal). **Kelsey Reider:** conceptualization (lead), data curation (lead), formal analysis (equal), investigation (supporting), methodology (equal), project administration (lead), supervision (lead), writing – original draft (equal).

## Conflicts of Interest

The corresponding author confirms on behalf of all authors that there have been no involvements that might raise the question of bias in the work reported or in the conclusions, implications, or opinions stated.

## Data Availability

The data that support the findings of this study are openly available in Open Science Framework at https://osf.io/gc57h/?view_only=b7934f57efd94f6e85242fc103decfe6.
